# Protection against Mucosal SHIV Challenge by Peptide and Helper-Dependent Adenovirus Vaccines

**DOI:** 10.3390/v1030920

**Published:** 2009-11-10

**Authors:** Eric A. Weaver, Pramod N. Nehete, Bharti P. Nehete, Stephanie J. Buchl, Donna Palmer, David C. Montefiori, Philip Ng, K. Jagannadha Sastry, Michael A. Barry

**Affiliations:** 1 Department of Internal Medicine, Division of Infectious Diseases, Translational Immunovirology Program, Mayo Clinic, Rochester, MN 55905, USA; 2 Department of Veterinary Sciences, M.D. Anderson Cancer Center, The University of Texas, Bastrop, TX 78602, USA; 3 Department of Molecular and Human Genetics, Baylor College of Medicine, Houston, TX 77030, USA; 4 Duke University Medical Center, Durham, NC 27710, USA; 5 Department of Immunology, M.D. Anderson Cancer Center, The University of Texas, Houston, TX 77054, USA; 6 Department of Immunology, Mayo Clinic, Rochester, MN 55905, USA; 7 Department of Molecular Medicine, Mayo Clinic, Rochester, MN 55905, USA

**Keywords:** HIV-1, SHIV, adenovirus, helper-dependent vector, mucosal challenge, serotype-switching

## Abstract

Groups of rhesus macaques that had previously been immunized with HIV-1 envelope (env) peptides and first generation adenovirus serotype 5 (FG-Ad5) vaccines expressing the same peptides were immunized intramuscularly three times with helper-dependent adenovirus (HD-Ad) vaccines expressing only the HIV-1 envelope from JRFL. No gag, pol, or other SHIV genes were used for vaccination. One group of the FG-Ad5-immune animals was immunized three times with HD-Ad5 expressing env. One group was immunized by serotype-switching with HD-Ad6, HD-Ad1, and HD-Ad2 expressing env. Previous work demonstrated that serum antibody levels against env were significantly higher in the serotype-switched group than in the HD-Ad5 group. In this study, neutralizing antibody and T cell responses were compared between the groups before and after rectal challenge with CCR5-tropic SHIV-SF162P3. When serum samples were assayed for neutralizing antibodies, only weak activity was observed. T cell responses against env epitopes were higher in the serotype-switched group. When these animals were challenged rectally with SHIV-SF162P3, both the Ad5 and serotype-switch groups significantly reduced peak viral loads 2 to 10-fold 2 weeks after infection. Peak viral loads were significantly lower for the serotype-switched group as compared to the HD-Ad5-immunized group. Viral loads declined over 18 weeks after infection with some animals viremia reducing nearly 4 logs from the peak. These data demonstrate significant mucosal vaccine effects after immunization with only env antigens. These data also demonstrate HD-Ad vectors are a robust platform for vaccination.

## 1. Introduction

The development of an effective HIV-1 vaccine is essential for controlling the HIV-1 pandemic. However, this goal has been difficult to accomplish due to the inherent biology of the virus including, but not limited to its propensity to infect immune cells and undergo high rates of mutation. A number of vaccine approaches are being developed to elicit these responses including live/attenuated HIV or SIV [1,2]; viral vectors including pox-, alpha-, and adenovirus vectors [3–7]; genetic immunization [8–10], peptide vaccines [11,12], and virus-like particle (VLP) vaccines [13–16].

It has been estimated that as much as 90% of HIV-1 infections occur by sexual transmission. In these cases, infection is thought to occur in most cases at vaginal, rectal, and urethral mucosal surfaces (reviewed in [17]). Given that the mucosal surface is the predominant entry route for HIV-1, there has been increasing interest in the development of vaccines that can generate robust antibody and cellular responses at mucosal surfaces (reviewed in [18]). Despite the recognized need for mucosal protection, most non-human primate challenge models involve intravenous injection of SIV or SHIV into animals. While this is appropriate to test the quality of systemic vaccination, this vaccine-challenge may not address whether mucosal protection is produced.

Adenoviral (Ad) vectors are one of the most robust gene-based vaccine vectors available [19–24]. Until recently, most adenoviral vaccine experiments have utilized the well-studied human adenovirus serotype 5 Ad (Ad5). While this virus is one of the most robust at generating anti-HIV immune responses, the majority of the human population has been exposed to this virus and have pre-existing neutralizing antibodies that can attenuate vaccine delivery [25]. While pre-existing antibodies are a problem, once an Ad vaccine is introduced into a non-immune host, this itself will provoke an anti-vector response that will quench subsequent use of this vaccine.

One approach to evade neutralizing antibodies is to “serotype-switch” the vector by changing the serotype of the Ad vaccine at each administration [26,27]. When applied for HIV vaccines, serotype-switching evades vector-induced immunity allowing robust prime-boost vaccination with different adenoviruses [28–32].

In most cases, Ad serotype-switching has been performed using first generation adenoviral (FG-Ad) vectors. We recently demonstrated proof of principle for the use of helper-dependent adenoviral (HD-Ad) vectors for serotype-switching in mice and non-human primates [33]. In HD-Ad vectors, all viral sequences are deleted from the vector with the exception of the inverted terminal repeats (ITRs) and packaging signal needed to replicate and package the vector. This allows sequences as large as 35 kilobase pairs to be packaged [34,35]. Because all adenoviral genes have been removed from the vector, no Ad proteins are expressed after vector delivery. Therefore, HD-Ad vectors generate lower vector-specific immune responses [36–38].

The HD-Ad system easily allows serotype switching, since Ads in the same species can cross-package each other’s genomes. We recently utilized species C Ad helper viruses from serotypes 1, 2, 5, and 6 to cross-package HD-Ad5 vectors expressing reporter genes or HIV-1 env [33]. By this approach, we demonstrated the HD-Ad vectors generated lower anti-vector immune responses and allowed multiple rounds of prime-boost against HIV-1 env in mice and in FG-Ad5-immune rhesus macaques [33].

In this work, we have mucosally challenged these HD-Ad-immunized macaques by rectal administration of the CCR5-tropic virus SHIV-SF162P3 [39]. We provide data on T cell and neutralizing antibody immune responses to complement our previous report on ELISA antibody responses against env. We also provide data on the effects on viral loads in the animals by repeated HD-Ad5 vaccination versus serotype-switch HD-Ad6, 1, and 2 vaccination.

## 2. Results and Discussion

### 2.1. Immunizations Prior to HD-Ad Vaccinations

**Figure 1.**
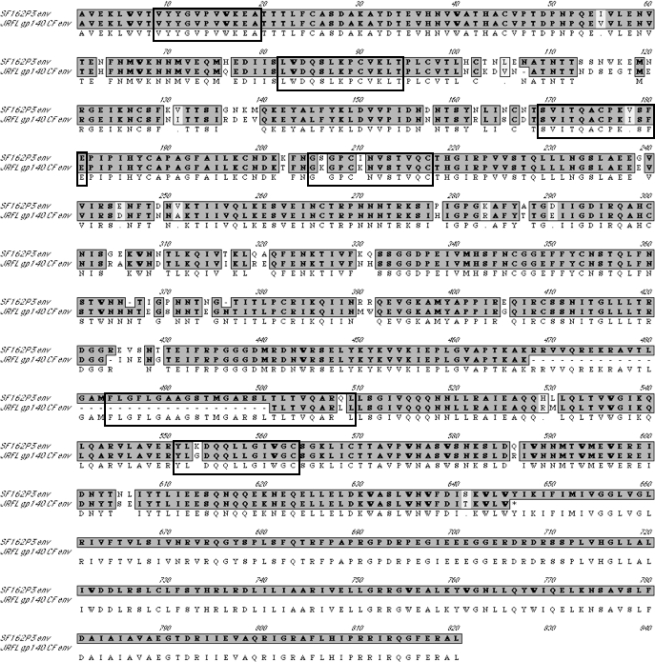
Protein sequence alignment of envelope antigens used in this study. The JRFL gp140 immunogen expressed by the HD-Ad vectors was aligned to the SF162P3 env protein of the challenge virus. Boxes indicate the locations of the six env peptides that were used to vaccinate the macaques prior to HD-Ad vaccination.

Eight macaques from previous studies (Table 1) were used in these experiments to conserve animals and for their prior immunizations with FG-Ad5 vectors. These animals had originally been immunized with various formulations of a synthetic peptide vaccine consisting of six conserved epitopes in the envelope (env) protein that have previously been shown to be effective at priming HIV-specific cellular immune responses in multiple animal models and humans [11,40–44]. These six peptides shown aligned to env in Figure 1 generate CD4 and CD8 responses without generating antibody responses. These peptides in various formulations mediate protection in macaques vs. SHIV-KU2 and SHIV-89.6P [43,44]. Prior to the HD-Ad study, macaques Rh51, Rh55, Rh62, and Rh63 had been vaccinated with the six synthetic peptides adjuvanted with FLT-3 ligand, CpG and by loading on dendritic cells (Table 1). Macaques Rh52, Rh61, Rh66, and Rh67 received a similar course with the exception of receiving an inactivated cholera toxin adjuvant (CT2*) rather than FLT-3 ligand and CpG (Table 1).

These animals were selected for study with HD-Ad, since they had all previously been vaccinated twice by the nasal route with 10^11^ virus particles (v.p.) of FG-Ad5 expressing a fusion protein of the six peptides (vector described in [45]). Therefore, these animals represented an Ad5 pre-immune population on which to test the utility of HD-Ad serotype-switching. While this was advantageous, prior immunizations with the six env peptides could affect T cell responses against these six epitopes that might be generated by the HD-Ad vaccines, but should not affect T cell responses outside these regions (Figure 1). Likewise, since the peptide vaccines do not generate antibodies against env, they would not be expected to confuse antibody effects of the HD-Ad vaccines.

### 2.2. HD-Ad Vaccinations

The macaques in this study were only vaccinated with env immunogens. No gag, pol, or other SHIV sequences were used. The JRFL gp140 env antigen in the Ad vaccines was generated by deletion of the furin cleavage site between gp120 and gp41 and deletion of the transmembrane domain. This immunogen therefore does not immunize against epitopes that are present in the cleavage and transmembrane domains in the SHIV-SF162P3 challenge virus. Alignment of the JRFL immunogen with the SF162P3 antigen shows 545 identical amino acids and 47 divergent amino acids within the common peptide sequences (Figure 1). Therefore, JRFL immunogen has 89% identity with the challenge virus.

Macaques Rh51 and Rh55 from the FLT group and animals Rh52 and Rh61 from the CT2* group were utilized for HD-Ad5 vaccination (Table 1). Monkeys Rh62 and Rh63 from the FLT group and macaques Rh66 and Rh67 from the CT2* group were used for HD-Ad6, 1, and 2 vaccination. Each group of four macaques were immunized at days 0, 24, and 67 with 10^11^ vp of the indicated HD-Ads expressing the JRFL gp140 form of env (Figure 1) by i.m. injection. Group 1 received HD-Ad5 three times. Group 2 received HD-Ad6, then HD-Ad1, then HD-Ad2 at the same time points.

### 2.3. Neutralizing Antibodies Generated Against HIV-1 Envelope

We previously reported on the antibody responses against env by ELISA [33]. This work revealed that FG-Ad5-immune animals that were immunized with only HD-Ad5-Env generated only minimal responses. In contrast, immunization with HD-Ad6-Env, HD-Ad1-Env, and HD-Ad2-Env generated detectable anti-env antibodies at each immunization with final antibody levels being 10-fold higher than in the HD-Ad5 group (p < 0.01) [33]. Given the high ELISA antibody responses, the samples were sent to the Immune Monitoring Core supervised by Dr. David Montefiori at Duke University to assess if these antibodies could neutralize SHIV or HIV viruses *in vitro* (Table 2). By this assay, only slight neutralization titers were observed when the samples were tested against SHIV-SF162P4 viruses and 89.6P.18, but not against other test viruses. Other field isolates tested were: SHIV-SF162P3.5, JRFL/293T, 6535.3, QH0692.42, SC422661.8 and PVO.4.

### 2.4. Neutralizing Antibodies Against Adenovirus

**Figure 2.**
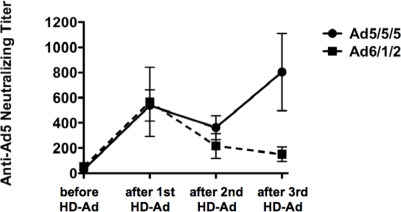
Neutralizing Antibody Responses Against Ad. Plasma samples taken at the indicated times were incubated with Ad5 expressing luciferase for 1 hour at 37°C prior to addition to A549 cells. 24 hours later, luciferase activity was measured and gene delivery was compared to untreated Ad5 vector. Data is expressed as geometric mean titers that reduced Ad luciferase activity 50%.

Ad5 neutralizing antibody levels were monitored in the animals after each immunization (Figure 2). Before first HD-Ad immunization, Ad5 neutralizing titers were 28 for the HD-Ad5 group and 52 for the serotype-switch group. This demonstrated that the prior intranasal FG-Ad5 immunizations had produced anti-Ad5 immunity in the animals. After first HD-Ad immunization, HD-Ad5 and HD-Ad6 boosted Ad5 neutralization titers to 500 in both groups. Two more immunizations with HD-Ad5 increased final titers to 800. One immunization with HD-Ad1 and then one with HD-Ad2 produced declining anti-Ad5 antibody levels that were three-fold lower than those generated by three HD-Ad5 immunizations. These data indicate that other viruses in species C can boost common neutralizing antibody levels (*i.e.* HD-Ad6), but that serotype-switching ultimately reduces the level of neutralizing antibodies after three immunizations.

### 2.5. T Cell Responses Generated by the HD-Ad Vaccines

PBMCs were harvested before and after each vaccination to monitor T cell responses against the env antigen by ELISPOT. PBMCs were stimulated either with the six epitopes of the peptide vaccine that was delivered prior to Ad vaccination or with overlapping 15-mer peptide pools from HIV-1 SF162P3 env covering the gp140 region in the HD-Ad vectors. Alignment of the JRFL gp140 immunogen with SF162P3 peptide pools shows 89% identity with the peptides used for ELISPOT. Alignment with of JRFL with the peptide vaccine shows amino acid mismatches in four of the six peptides (Figure 1).

**Figure 3.**
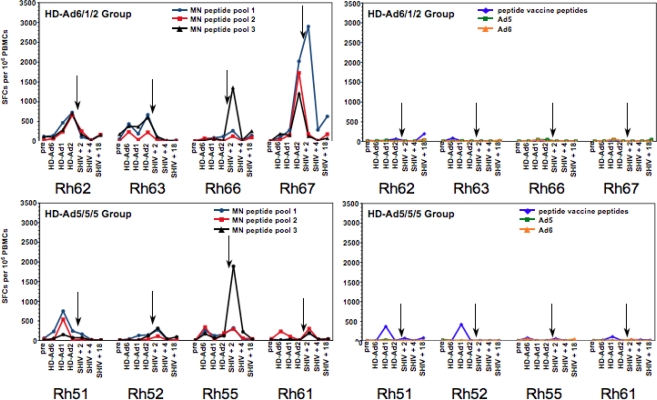
IFN-γ ELISPOT of PBMCs from macaques during HD-Ad vaccination and after SHIV challenge. PBMCs were stimulated with SF162P3 env peptide pools, the six conserved env peptides, or Ad5 or Ad6 viruses. Spot forming cells (SFC) as measured by ELISPOT are shown relative to the y axis, with the time point of assay before and after vaccination and challenge shown below each graph. The HD-Ad5 group is shown in lower panels and the serotype-switched (HD-Ad6, 1, 2) group is shown in the top panels. On the x-axis, HD-Ad designates time points 2 weeks after each vaccination. Arrows indicate the time of SHIV challenge. SHIV+2, SHIV+4, and SHIV+18 designate weeks 2, 4, and 18 after SHIV challenge.

ELISPOT testing before HD-Ad vaccination revealed responses below background for two macaques in the HD-Ad6/1/2 group and three in the HD-Ad5 group (Figure 3). The three other macaques had weak ELISPOT signals of 200 or less SFCs per 10^6^ cells (Figure 3). With each HD-Ad immunization, CD8-IFN-γ SFCs generally increased in both groups when after stimulation with the SF162P3 env overlapping peptide pools. Responses were higher against the SF162P3 peptides in all of the serotype-switched animals and were less variable than in the HD-Ad5 group. T cell responses peaked after one or two immunizations in the HD-Ad5 group with peaks from 200 to 800 SFCs per 10^6^ cells. In contrast, T cell responses peaked in most serotype-switched animals after third immunization with highest SFCs ranging from 700 to 2,000 SFCs (Table 3). When the six peptides of the peptide vaccine were used to stimulate the PBMCs, SFC responses in both groups were substantially lower and less frequent (Figure 3), suggesting that most of the T cell responses were directed at epitopes outside those covered by the peptide vaccine (Figure 1). Stimulation of the PBMCs with Ad5 or Ad6 produced largely undetectable T cell responses suggesting responses were predominantly against the env immunogen rather than against the Ad vectors.

### 2.6. Mucosal SHIV Challenge

**Figure 4.**
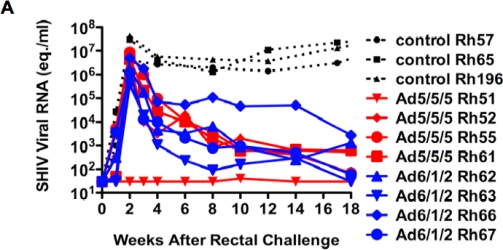

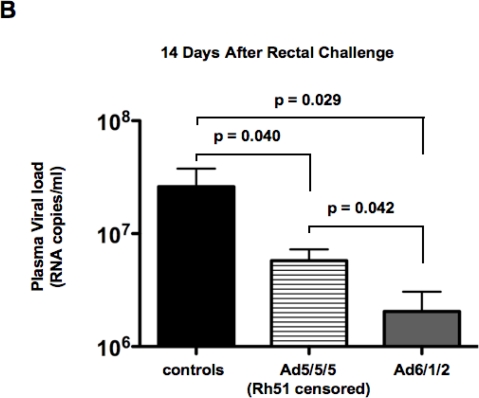
Plasma viral loads after rectal SHIV-SF162P3 challenge. Three control macaques and the eight HD-Ad vaccinated macaques were challenged rectally by atraumatic administration of 1,000 TCID_50_ of SHIV-SF162P3. Viral loads were assessed by quantitative realtime PCR of viral genomes from the blood at the indicated times after challenge. **A)** Viral loads over 18 weeks after challenge. **B)** Comparison of peak viral titers at 2 weeks after challenge. Rh51 was excluded from the analysis due to high probability that the animal was not infected with SHIV rather than sterilizing immunity was generated.

To mimic sexual transmission of HIV, macaques were challenged by atraumatic administration of 1,000 TCID_50_ of the CCR5-tropic virus SHIV-SF162P3 (Figure 4). Challenge of three control macaques produced peak viremia within 2 weeks with viral loads above 2×10^7^ viral genomes per ml of plasma (Figure 4B). Viral loads remained above 10^6^ copies/ml for 6 months at which time two of the animals were sacrificed due to weight loss and AIDS-like symptoms.

The HD-Ad-immunized animals were challenged 4 months after first immunization. This SHIV-SF162P3 challenge produced lower peak viremia and viral set points (Figure 4). At peak, viral loads were 2 to 10-fold lower in the HD-Ad vaccinated group than in control animals. One animal Rh51 in the HD-Ad5 group had viral RNA levels below detection and so appeared to have not had a “take” of the challenge virus. When Rh51 was censored, peak viremia at 2 weeks for both vaccine groups was significantly lower than controls (p = 0.04, Figure 4B). Notably, peak viremia for the HD-Ad6/1/2 group was significantly lower than the HD-Ad5/5/5 group (p < 0.05). By 18 weeks, viral set points were below 3,000 copies for all of the immunized macaques. This was notable, since all of these animals were only vaccinated with env antigens. No gag or other SHIV antigens were used. While the HD-Ad5/5/5 and HD-Ad6/1/2 groups were not significantly different from each other at this time, it was interesting that that the viral RNA levels for Rh55 from the HD-Ad5 group and Rh63 and Rh67 from the HD-Ad6/1/2 group were down to 30–60 eq./ml or 4 orders of magnitude down from their peak viremia.

### 2.7. Discussion

We previously reported the use of HD-Ad vectors for HIV vaccination [33]. In this earlier work, we were able to utilize eight macaques that had previously been immunized nasally with FG-Ad5 to test our ability to vaccinate in Ad5-immune macaques. We demonstrated that serotype-switching did indeed provide robust circumvention of pre-existing immunity in these non-human primates and allowed the production of anti-env antibody responses that were 9 times higher than those generated by HD-Ad5 vectors [33].

In this work, we have analyzed the production of neutralizing antibodies against the env transgene protein and against Ad5 itself. This work shows that the strong anti-env ELISA titers that we observed after serotype-switching unfortunately did not translate into the production of robust neutralizing antibodies against HIV or SHIV. These data suggest that protection was mediated by T cell responses or by other antibody mechanisms (*i.e.* antibody-dependent cellular cytotoxicity (ADCC) [46], *etc.*). This is consistent with previous observations that SHIV-SF162P3 is notoriously hard to neutralize with antibodies [47,48].

While these Ad5 pre-immune animals provided a good model to test for antibody production alone, they had also been previously been vaccinated with the six env peptides in various formats (Table 1). Since these peptide vaccines do not generate antibody responses, this did not affect comparison of env antibody production by the HD-Ad vaccines, but could affect the production of T cell responses by acting as priming vaccines for the HD-Ad vaccines. To test this, we compared PBMC ELISPOT responses against the cognate six epitopes used in the previous vaccinations and against overlapping 15-mer peptides from SF162P3 env spanning the vaccine’s gp140 region. This comparison revealed that there was little cross-reactivity generated by the HD-Ad vaccines against the six peptides, but stronger T cell responses were generated against the peptide pools. These data suggest that the HD-Ad vaccines are generating much of the detectable T cell responses observed in the macaques.

While prior immunization with the peptides complicated data analysis, in the interest of the strong responses we observed and to minimize future animal use, we opted to challenge these animals with SHIV. We performed mucosal challenge by the rectal route with the CCR5-tropic virus SHIV-SF162P3 to mimic sexual transmission of the virus. While SIV is arguably a more suitable mucosal challenge virus than SHIV, our challenge virus had to express an HIV-1 env to assess the HIV-1 env-directed immunity that the HD-Ad vaccines had established. This challenge demonstrated that control animals had severe peak viremia after mucosal challenge and that this viremia persisted for six months until AIDS-like symptoms necessitated euthanasia of two of the animals. In contrast to controls, the HD-Ad vaccinated animals had 2 to 10-fold lower peak viremia and viral loads generally trended downward over the next 4 months. For three of the animals, viral loads approached the limits of detection by 18 weeks. These data suggest that the peptide vaccines, the HD-Ad vaccines, or both lead to lower viral loads in the animals after mucosal challenge. Given the observed ELISPOT responses, we speculate that much of this protection was mediated by the Ad vaccines.

Comparison of the HD-Ad5/5/5-immunized animals and the serotype-switched HD-Ad6/1/2 group demonstrated that animals vaccinated with the different serotypes had statistically lower peak viremia than those immunized with only HD-Ad5. This confirms the utility of serotype-switching that has previously been observed using FG-Ad vectors [28–32]. This also suggests that some level of the protection against SHIV challenge was actually mediated by the Ad vectors rather than the earlier peptide vaccines, since the serotype-switched vaccine generated more robust immune responses that may have resulted in the lower peak and set point viral loads. Peak cellular responses in the HD-Ad6/1/2 serotype-switched group were observed after the third immunization for three of the four immunized animals. This indicates that serotype-switching was driving anamnestic responses while the HD-Ad5/5/5 group immune responses may have become senescent due to increasing anti-Ad5 neutralizing antibodies (Fig. 2 and Table 3). This comparison is based on censoring Rh51 from the analysis, since it had undetectable viral loads throughout the study. Censoring this animal is based on the assumption that the undetectable viral loads in HD-Ad5/5/5 group monkey Rh51 were due to poor “take” of the challenge virus. If Rh51 is included, the two groups are equal to each other by statistical comparison. While it is formally possible that the vaccine fully protected Rh51, we are unaware of an example of sterilizing immunity being generated by any vaccine in this model. In addition, T cell and antibody responses in Rh51 were comparable to those in other macaques. Therefore, the most likely explanation is that Rh51 merely was not robustly infected by the challenge virus.

## 3. Experimental Section

### 3.1. Adenoviruses

HD-Ad1, 2, 5, and 6 viruses expressing the gp140 form of HIV-1 JRFL were produced as previously described [33]. HD-Ad5-env vector was transfected into a 60-mm dish of Cre-expressing 116 cells expressing Cre recombinase as in [49]. The transfected cells were infected a day later with the E1-deleted Ad5 helper virus AdNG163 whose packaging signal is flanked by loxP sites [49] for deletion in the Cre cells. Lysates were subsequently amplified by serial infections with AdNG163 in 116 cells. CsCl-banded HD-Ad were then produced from 3 liters of 116 cells producing HD-Ad preps with E1 -deleted helper contamination less than 0.02% [49]. HD-Ad1, 2, and 6 vectors were generated with helper viruses Ad1LC8cCEVS-1, Ad2LC8cCARP [26], and Ad6LC8cCEVS-6, respectively that were generously provided by Carole Evelegh and Frank L. Graham (McMaster University).

### 3.2. Animals

All animal experiments were carried out according to the provisions of the Animal Welfare Act, PHS Animal Welfare Policy, and the principles of the NIH Guide for the Care and Use of Laboratory Animals, and the policies and procedures of the University of Texas MD Anderson Cancer Center. Eleven adult male rhesus macaques (*Macaca mulatta*) of Indian origin were maintained in the specific pathogen-free breeding colony at the Michael Keeling Center for Comparative Medicine and Research of The University of Texas MD Anderson Cancer Center, Bastrop TX. The animals were anesthetized during procedures to minimize discomfort. The animals were not screened for Mamu genotype prior to study, but were randomized into the two HD-Ad vaccine groups based to equally segregate animals previously treated into both groups.

### 3.3. Immunizations Prior to HD-Ad Vaccinations

Eight macaques from previous studies (Table 1) were used in these experiments. These animals had originally been immunized with various adjuvanted synthetic peptide vaccines consisting of six conserved env epitopes [11,40–44].

### 3.4. HD-Ad Vaccination

These peptide and FG-Ad5-immunized macaques were immunized at days 0, 24, and 67 with 10^11^ vp of the indicated HD-Ads by i.m. injection (Table 1).

### 3.5. Collection of Samples

Samples were collected at each time point indicated before any immunization or procedure. Peripheral venous blood samples were collected in EDTA or sodium heparin. Before the separation of peripheral blood mononuclear cells (PBMC) from the blood samples, plasma was separated and stored immediately at −80°C. Peripheral Blood Mononuclear Cells (PBMCs) were prepared from the blood on Ficoll-Hypaque density-gradients.

### 3.6. Assay for Neutralization of HIV and SHIV

Neutralization was measured as a reduction in luciferase reporter gene expression after a single round of infection in TZM-bl cells as described [50,51]. TZM-bl cells were obtained from the NIH AIDS Research and Reference Reagent Program, as contributed by John Kappes and Xiaoyun Wu. Briefly, 200 TCID_50_ of virus was incubated with serial 3-fold dilutions of test sample in duplicate in a total volume of 150 μl for 1 hr at 37°C in 96-well flat-bottom culture plates. Freshly trypsinized cells (10,000 cells in 100 μl of growth medium containing 75 μg/ml DEAE dextran) were added to each well. One set of control wells received cells + virus (virus control) and another set received cells only (background control). After a 48 hour incubation, 100 μl of cells was transferred to a 96-well black solid plates (Costar) for measurements of luminescence using the Britelite Luminescence Reporter Gene Assay System (PerkinElmer Life Sciences). Neutralization titers are the dilution at which relative luminescence units (RLU) were reduced by 50% compared to virus control wells after subtraction of background RLUs. Assay stocks of molecularly cloned Env-pseudotyped viruses were prepared by transfection in 293T cells and were titrated in TZM-bl cells as described [50]. The clade B reference Env clones were described previously [50].

### 3.7. Assay for Neutralization of Ad5

Ad5 neutralization was performed as described previously [45]. Briefly, serial dilutions of plasma were incubated in triplicate for 1 hour at 37°C with Ad5 vector expressing luciferase. The resulting solution was added to A549 cells for 24 hours and luciferase activity was measured. Data is expressed as geometric mean titers that reduced Ad luciferase activity 50%.

### 3.8. ELISPOT assay for detecting antigen-specific IFN-γ producing cells

Freshly prepared PBMC were used for the IFN-γ ELISPOT assay as described previously [52]. PBMCs were either stimulated with synthetic peptides pools, with Ad expressing env, or with Con A (5 μg/ml) as positive control reagent. For the six peptide vaccine cocktail, the six epitopes (Figure 1) were mixed as a pool. For overlapping envelope peptides, the SF162P3 env 15-mer peptide set (NIH AIDS Reagent Program) was used as 3 pools of 50 to 70 peptides spanning the gp140 region. Alignment of the JRFL immunogen encoded in the Ad vectors with SF162P3 peptide pool shows 89% identity with the peptide pool used for ELISPOT. PBMCs (1 × 10^5^) were seeded in duplicate wells of 96-well plates (polyvinylidene difluoride backed plates, MAIP S 45, Millipore, Bedford, MA) coated with anti-IFN-γ. The cells were incubated in the presence of the various antigens for 36 h at 37°C. The cells were then removed, the wells washed, and then incubated with 100 μl of biotinylated anti-IFN-γ for 3 h at 37°C followed by avidin-HRP for another 30 minutes. Spots representing individual cells secreting IFN-γ were developed using 0.3 mg/ml of 3-amino-9-ethyl-carbazole in 0.1 M sodium acetate buffer, containing 0.015% hydrogen peroxide. The plates were washed to stop development and the spots were counted by an independent agency (Zellnet Consulting, New Jersey, NJ). The responses in terms of IFN-γ spot forming cells (SFC) for 10^5^ total input CD8^+^ T cells were determined for individual monkeys after subtracting background values of cells cultured in the medium. The cut off value for determining the positive response in the assay is defined as a minimum of 10 spots that is twice the number observed in cells cultured in the medium. Data is represented as SFCs per 10^6^ PBMCs for comparison to previous reports in the literature.

### 3.9. Virus Challenge

Macaques were challenged macaques by intrarectal inoculation of 1,000 TCID_50_ of SHIV-SF162P3 from the NIH AIDS Reagent Program.

### 3.10. Viral Load Determination

SHIV viral loads from the blood were determined by determining viral RNA copy numbers by real-time RT-PCR analyses. These assays were performed at the NIH Core Facility by Dr. Jeff Lifson’s group. The threshold sensitivity of the assay is 30 viral RNA copy-equivalents/ml of plasma, and the inter-assay variation is <25% (coefficient of variation).

### 3.11. Statistical Analyses

Data was evaluated using GraphPad Prism 4 software. P values ≤ 0.05 were considered statistically significant.

## 4. Conclusions

This study demonstrates that serotype-switched HD-Ad vaccines generate higher immune responses and lower viral loads after mucosal challenge with a CCR5-tropic SHIV. This provides proof of principle for applying these vaccines systemically or mucosally to repel mucosal entry by SIV or HIV-1. These data are also notable given the fact that these non-human primates were only immunized with the envelope immunogen. No gag, pol, nef, or other SIV or HIV proteins we used for vaccination. This suggests that delivery of these missing lentiviral antigens by HD-Ad vaccines may well provide even more substantial protection against mucosal challenge.

## Figures and Tables

**Table 1.** Vaccines Used in the Macaques in this Study.

Macaque	Env peptides + FL3L + CpG 3X 12/2006	Env peptides + CT2* 3X 12/2006	Ad-EnvPep nasal 2X 2/2007	Env Peptides + DCs 2X 3/2008	HD-Ad5-Env 3X 9/2008	HD-Ad6-Env HD-Ad1-Env HD-Ad2-Env 9/2008
Rh51	+		+	+	+	
Rh55	+		+	+	+	
Rh52		+	+	+	+	
Rh61		+	+	+	+	
Rh62	+		+	+		+
Rh63	+		+	+		+
Rh66		+	+	+		+
Rh67		+	+	+		+

Dates shown designate when each vaccine was applied to the indicated animals.

**Table 2.** Neutralizing Antibodies vs. SHIV.

		ID50 in TZM-bl cells^1^	

Animal	Bleed day	SHIV-SF162P4 (ID#762)	SHIV-89.6P.18 (ID#767)
51	0	<20	<20
	24	<20	<20
	57	<20	<20
	83	<20	<20

52	0	36	20
	24	25	<20
	57	<20	<20
	83	25	<20

55	0	31	<20
	24	23	<20
	57	<20	<20
	83	55	23

61	0	25	<20
	24	<20	<20
	57	22	<20
	83	<20	<20

62	0	<20	<20
	24	<20	<20
	57	<20	<20
	83	<20	<20

63	0	25	<20
	24	<20	<20
	57	<20	<20
	83	25	<20

66	0	<20	<20
	24	<20	<20
	57	<20	<20
	83	22	<20

67	0	<20	<20
	24	<20	<20
	57	<20	<20
	83	46	<20

1 Values are the sample dilution at which relative luminescence units (RLUs) were reduced 50% compared to virus control wells (no test sample).

**Table 3.** Total anti-SF162P3 ELISPOT responses.

	HD-Ad5/5/5 Group				HD-Ad6/1/2 Group			
	**Rh51**	**Rh52**	**Rh55**	**Rh61**	**Rh62**	**Rh63**	**Rh66**	**Rh67**
	
Post-1	200	30	**730**	**170**	50	810	140	300
Post-2	**1340**	120	160	80	730	350	**190**	570
Post-3	220	**260**	440	0	**1820**	**1250**	120	**4940**
	
Post-SHIV	0	80	50	0	230	0	480	860

Values represent the combined ELISPOT responses to all 3 pools of overlapping peptides with pre-HD-Ad immune responses subtracted. Bold values indicate peak cellular anti-SF162P3 immune responses.
